# Antimicrobial resistance and molecular genotyping of *Salmonella enterica* serovar Enteritidis clinical isolates from Guizhou province of Southwestern China

**DOI:** 10.1371/journal.pone.0221492

**Published:** 2019-09-23

**Authors:** Xiaoyu Wei, Lv You, Dan Wang, He Huang, Shijun Li, Dingming Wang

**Affiliations:** 1 Laboratory of Bacterial Infectious Disease of Experimental Center, Guizhou Provincial Center for Disease Control and Prevention, Guiyang, China; 2 Institute of Communicable Disease Control and Prevention, Guizhou Provincial Center for Disease Control and Prevention, Guiyang, China; Instiuto Ramon y Cajal de Investigacion Sanitaria (IRYCIS), SPAIN

## Abstract

*Salmonella enterica* serovar Enteritidis (*S*. Enteritidis) is the most common and essential serotype that causes salmonellosis in Guizhou province. This study aimed to investigate the antimicrobial resistance (AMR) and molecular genotyping of 79 *S*. Enteritidis clinical isolates from 2011 to 2016 in Guizhou, China. Antimicrobial resistance and minimum inhibitory concentrations (MICs) of *S*. Enteritidis clinical isolates were detected by micro broth dilution method against ten classes 16 antimicrobial agents, and molecular genotyping were examined by pulsed-field gel electrophoresis (PFGE) and multiple-locus variable-number tandem repeat analysis (MLVA). All (100%) isolates showed resistance to at least one antimicrobial. Resistance to nalidixic acid (98.7%) was the highest, followed by sulfamethoxazole (87.3%) and ampicillin (77.2%). The majority of isolates (92.4%) showed decreased susceptibility to ciprofloxacin. Resistance to the third and fourth-generation cephalosporins was observed. Twenty-six AMR profiles were observed, and the predominant AMR profile was ampicillin-streptomycin-sulfamethoxazole-amoxicillin/clavulanic acid-nalidixic acid. A high burden of multidrug resistance (MDR) (81.0%) was found. Seventy-nine *S*. Enteritidis isolates were divided into 33 different pulsotypes (PTs), and the most frequent PT was PT18. Twenty-six different MLVA types (MTs) were generated with seven VNTR loci analysis of these isolates. The dominant PTs and MTs were persistent during 2011–2016. *S*. Enteritidis clinical isolates showed higher genetic diversity using PFGE combined with MLVA grouped into 60 PT-MT genotypes. No correlation was observed between genotypes, AMR profiles and geographic location. These data revealed the characteristics of AMR and molecular genotyping of *S*. Enteritidis clinical isolates in Guizhou province. These results highlight that strengthening the AMR and molecular genotyping surveillance is essential to prevent and control salmonellosis in Guizhou. PFGE combined with MLVA should be powerful tools for the molecular genotyping of *S*. Enteritidis isolates.

## Introduction

Nontyphoidal *Salmonella* (NTS) is one of the most common pathogens that contaminate food and causes diarrhea in humans. 93.8 million cases of gastroenteritis due to *Salmonella* species occur each year globally, with 155,000 deaths [1]. In China, 9.87 million cases of gastroenteritis caused by *Salmonella* each year [2], and outbreak events due to *Salmonella* infection sometimes occurred [3]. NTS was the dominant pathogen, which caused microbiological foodborne outbreaks with the largest number of cases in China [3]. *Salmonella enterica* serovar Enteritidis (*S*. Enteritidis) is the most common serotype among human isolates globally [4]. A recent survey showed NTS was the top five pathogens causing diarrhea in children under age five and elderly outpatients older than 65 years in China [5, 6], and of all identified NTS isolates, *S*. Enteritidis was the most common serotype [5, 6].

Molecular genotypes can be helpful to active outbreak investigation and surveillance of pathogen, especially same serotype isolates. PFGE is a standard and international molecular subtyping method to discern *Salmonella* strains and often used for surveillance and outbreak investigations [7]. Although PFGE is considered as the gold-standard for subtyping *Salmonella*, it is time-consuming, labor-intensive and offers low throughput [8]. The discriminatory power of single endonucleotidase analysis of PFGE is limited for outbreak detection of *S*. Enteritidis [9]. MLVA is another molecular genotyping method used by microbiologists to generate a DNA fingerprint, which is based on a fragment size analysis of the number of repeats in the variable number tandem repeats (VNTR) region of microbial genome in most bacterial species. MLVA is usually examined after PFGE has been performed. Meanwhile, MLVA genotyping method showed much higher discriminatory power than PFGE in studies from other countries [10, 11]. The use of PFGE combined with MLVA is suitable for efficiently genotyping *S*. Enteritidis strains and provides crucial epidemiological information [12].

Guizhou is a multinational and mountainous area in the southwest of China with 34.75 million permanent residents, which is known as a “natural encyclopedia” of the karst landform. Recent years, rich natural, cultural and environmental resources have been to boost tourism in Guizhou, which may permit the dissemination of diarrhea pathogens and AMR. Our previous surveillance on bacterial pathogens of diarrheal patients in Guizhou province showed that NTS was the primary pathogen caused infectious diarrhea and *S*. Enteritidis was the most common serotype [13–15]. Recently, several foodborne outbreaks caused by *S*. Enteritidis had been reported in Guizhou [16–18]. Wide-ranging drug resistance and a high proportion of MDR in the scaled farm, pork and chicken were reported in Guizhou [19–21].

However, data on AMR and molecular genotyping of *S*. Enteritidis clinical isolates from Guizhou have lacked so far. This study aimed to perform AMR, PFGE and MLVA of *S*. Enteritidis clinical isolates from Guizhou for better understanding the AMR and molecular epidemiologic characteristics and revealing associations between AMR profiles, genotypes and geographic location.

## Methods

### Ethics statement

The present study was reviewed and approved by the Ethics Review Committee of Guizhou Provincial Center for Disease Control and Prevention. All data/isolates were analyzed anonymously.

### Bacterial isolates and identification

Bacterial isolates used in this study were derived from clinical patients (stool and blood) from 2011 to 2016 in Guizhou province, Southwest of China. All 79 *S*. Enteritidis strains were isolated from eight cities (Anshun, n = 14; Guiyang, n = 23; Qiandongnan, n = 3; Qiannan, n = 2; Qianxinan, n = 10; Tongren, n = 6; Zunyi, n = 19; Bijie, n = 2). All isolates were stored in 20% glycerol in -80°C, which were revived by inoculation into Luria-Burtani (LB) agar plates. These isolates were confirmed as *S*. Enteritidis with API20E identification kits (Biomerieux, France) and serotyped by slide agglutination with commercial *Salmonella* poly and monovalent O and H antisera (SSI, Denmark).

### Antimicrobial susceptibility test

The MICs for 79 *S*. Enteritidis isolates were determined using the micro broth dilution method according to the manufacturer’s instructions (Xingbai, Shanghai, China). It contained 16 animicrobial agents including ampicillin (AMP), amoxicillin/clavulanic acid (AMC), ceftriaxone (CRO), cefepime (FEP), cefoxitin (FOX), imipenem (IPM), gentamicin (GEN), streptomycin (STR), nalidixic acid (NAL), ciprofloxacin (CIP), sulfamethoxazole (SOX), trimethoprim /sulfamethoxazole (SXT), azithromycin (AZM), chloramphenicol (CHL), doxycycline (DOX) and tetracycline (TET) that are in ten classes of drugs (penicillin, β-Lactams, cephems, macrolides, carbapenems, aminoglycosides, quinolone and fluoroquinolones, tetracyclines, phenicols and sulfonamides). Briefly, freshly culture bacteria were suspended in saline and adjusted to a 0.5 Mcfarland turbidity standard concentration. The 60 μl bacterial suspension was absorbed into a disposable aseptic groove with a pipettor, then 12 ml nutrient broth was added into the groove and mixed intensively. 100 μl of the mixture was added into each hole of 96 holes microporous plate except a negative hole. The 100 μl nutrient broth was added into the negative hole. The microporous plates were incubated at 35°C for 18h. Isolates were classified as susceptible, intermediate or resistant according to the Clinical Laboratory Standards Institute guidelines (CLSI, 2017) except streptomycin. The interpretive standards of streptomycin used were the National Antimicrobial Resistance Monitoring System for enteric bacteria (NARMS) established breakpoints for *Salmonella* isolates (https://www.cdc.gov/narms/antibiotics-tested.html). The MIC of streptomycin used susceptible ≤16 μg/mL, no MIC range of intermediate susceptibility exists, and resistant ≥32 μg/mL. Decreased susceptibility to ciprofloxacin (MIC ≥0.12 μg/mL) includes isolates with MICs categorized as intermediate or resistant. *Escherichia* coli ATCC25922 was used as a control strain. Multidrug-resistant (MDR) was identified as resistance to three or more classes of drugs.

### Pulsed-field gel electrophoresis analysis

PFGE was performed according to the standard operating procedure for pulsenet PFGE of *Salmonella* serotypes (https://www.cdc.gov/pulsenet/pathogens/pfge.html) with CHEF DRIII (Bio-Rad, USA). All isolates, including the reference strain-*Salmonella* serovar Braenderup H9812 in this study, were digested with *Xba*I enzyme (New England Biolabs, Leusden, The Netherlands). The cluster analysis of PFGE was performed using BioNumerics software (Version7.6; Applied Maths), and the Dice coefficient was determined using the unweighted pair group method with arithmetic averages (UPGMA). Band comparison was performed using dice coefficient with 1.50% optimization and 1.50% position tolerance. If the PFGE patterns of isolates have the same numbers of bands and the same apparent size, isolates are designated genetically indistinguishable[22].

### Multiple-locus variable-number tandem repeat analysis

Laboratory standard operating procedure for pulsenet MLVA of *S*. Enteritidis was accessible on the website (https://www.cdc.gov/pulsenet/pathogens/mlva.html). The PCR primers of seven VNTR loci (SE1, SE2, SE3, SE5, SE6, SE8, and SE9) were synthesized according to the procedure by Biotechnology Corporation (Tianyi Huiyuan, Beijing, China). Capillary electrophoresis was performed for PCR products on an Applied Biosystems Genetic Analyzer 3730xl. The data were clustered with the categorical coefficient and generated a minimum spanning tree (MST) with BioNumerics software basing on seven VNTR loci to know the genetic relationship, which followed the laboratory standard operating procedure.

## Results

### Antimicrobial resistance and MICs of *S*. Enteritidis

Antimicrobial resistance testing showed that all 79 *S*. Enteritidis isolates exhibited resistance to at least one class antimicrobial in Guizhou (S1 Table). *S*. Enteritidis isolates were shown to be the most resistant to nalidixic acid (98.7%, 78/79), followed by the resistance to sulfamethoxazole (87.3%, 69/79), ampicillin (77.2%, 61/79), streptomycin (75.9%, 60/79) and amoxicillin/ clavulanic acid (49.4%, 39/79) (Table 1). Furthermore, *S*. Enteritidis isolates were shown to be resistant to the third and fourth-generation cephalosporins, including ceftriaxone (7.6%, 6/79) and cefepime (6.3%, 5/79). None of these isolates was resistant to cefoxitin. Only 3.8% (3/79) of *S*. Enteritidis isolates were resistant to ciprofloxacin. However, 92.4% (73/79) of isolates were shown to be decreased susceptibility to ciprofloxacin (MIC ≥0.12 μg/mL) (S1 Table). Additionally, imipenem and azithromycin resistance were detected in 1.3% (1/79) and 3.8% (3/79) of *S*. Enteritidis isolates, respectively. The MICs of each antimicrobial for 79 *S*. Enteritidis were displayed in Table 2.

**Table 1:** Antimicrobial resistance of 79 *S*. Enteritidis clinical isolates in Guizhou from 2011 to 2016.

Antimicrobial	Number of isolates (%)						
	2011 (n = 7)	2012 (n = 3)	2013 (n = 14)	2014 (n = 16)	2015 (n = 12)	2016 (n = 27)	Total (n = 79)
**Penicillin**							
Ampicillin (AMP)	5(71.4)	3(100)	10(71.4)	12(75)	10(83.3)	21(77.8)	61(77.2)
**β-Lactams**							
Amoxicillin/clavulanic acid (AMC)	4(57.1)	3(100)	8(57.1)	12(75)	10(83.3)	2(6.9)	39(49.4)
**Cephems**							
Ceftriaxone (CRO)	0	0	0	1(6.2)	2(16.7)	3(11.1)	6(7.6)
Cefepime (FEP)	0	0	0	1(6.2)	2(16.7)	2(7.4)	5(6.3)
Cefoxitin (FOX)	0	0	0	0	0	0	0
**Carbapenems**							
Imipenem (IPM)	0	0	0	0	0	1(3.7)	1(1.3)
**Aminoglycosides**							
Gentamicin (GEN)	3(42.9)	1(33.3)	0	1(6.2)	0	3(11.1)	8(10.1)
Streptomycin (STR)	4(57.1)	2(66.7)	11(78.6)	12(75)	11(91.7)	20(74.1)	60(75.9)
**Quinolones and Fluoroquinolones**							
Nalidixic acid (NAL)	7(100)	3(100)	14(100)	16(100)	12(100)	26(96.3)	78(98.7)
Ciprofloxacine (CIP)	2(28.6)	0	0	0	0	1(3.7)	3(3.8)
**Sulfonamides**							
Sulfamethoxazole (SOX)	5(71.4)	3(100)	13(92.9)	13(81.2)	12(100)	23(85.2)	69(87.3)
Trimethoprim /sulfamethoxazole (SXT)	2(28.6)	0	1(7.1)	5(31.2)	1(8.3)	4(14.8)	13(16.5)
**Macrolides**							
Azithromycin (AZM)	0	0	0	0	0	3(11.1)	3(3.8)
**Phenicols**							
Chloramphenicol (CHL)	0	0	0	1(6.2)	1(8.3)	0	2(2.5)
**Tetracyclines**							
Doxycycline (DOX)	2(28.6)	0	1(7.1)	6(37.5)	4(33.3)	6(22.2)	19(24.1)
Tetracycline (TET)	1(14.3)	0	1(7.1)	5(31.2)	4(33.3)	7(25.9)	18(22.8)
**Multi-drug resistance (MDR)**	5(71.4)	3(100)	11(78.6)	13(81.3)	11(91.7)	21(77.8)	64(81.0)

**Table 2:** MIC_50_ and MIC_90_ of 16 antibiotics for 79 *S*. Enteritidis clinical isolates.

antibiotics	MIC range	MIC_50_	MIC_90_
Ampicillin	1–64	64	64
Amoxicillin/clavulanic acid	1–64	16	64
Ceftriaxone	0.25–16	0.25	0.5
Cefepime	1–64	1	1
Cefoxitin	1–8	2	4
Imipenem	0.5–32	0.5	0.5
Gentamicin	1–64	1	32
Streptomycin	8–64	64	64
Nalidixic acid	4–64	64	64
Ciprofloxacine	0.03–8	0.125	0.25
Sulfamethoxazole	16–1024	512	512
Trimethoprim /sulfamethoxazole	0.13–8	0.25	8
Azithromycin	2–128	2	8
Chloramphenicol	2–128	8	16
Doxycycline	2–64	4	64
Tetracycline	2–64	4	64

**Fig 1:**
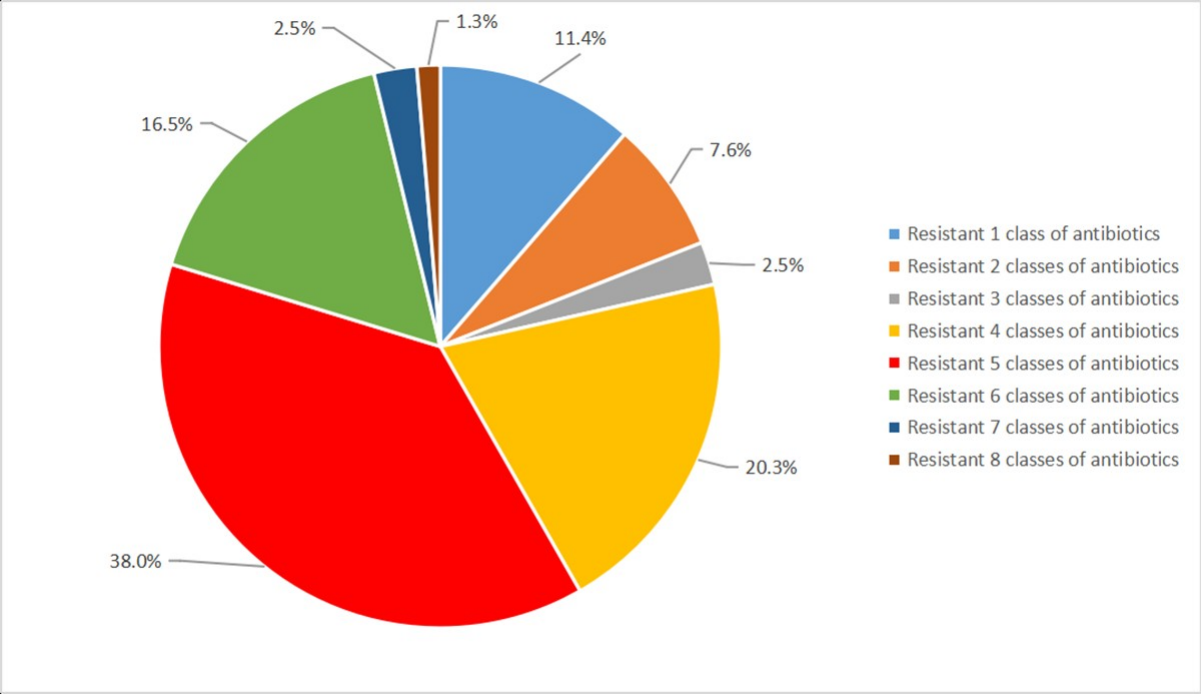
The percentage of resistance to ten classes antibiotics of *S*. Enteritidis isolates. Our analysis showed that resistance to five classes of antibiotics was the most frequent among 79 *S*. Enteritidis isolates.

In all isolates, high-level MDR *S*. Enteritidis isolates were observed. 81.0% (64/79) of *S*. Enteritidis isolates were resistant to three or more classes of antimicrobial agents. Resistance to five classes antibiotics was the most frequent, accounting for 38.0% (30/79) of *S*. Enteritidis isolates, whereas exhibited MDR to at most eight classes antibiotics (Fig 1). Furthermore, twenty-six AMR profiles were observed among *S*. Enteritidis isolates. The predominant AMR profile was ampicillin-streptomycin-sulfamethoxazole-amoxicillin/clavulanic acid-nalidixic acid (27.8%, 22/79). Meanwhile, 44.3% (35/79) of *S*. Enteritidis isolates were resistant to at least the combination of traditional antimicrobials, including ampicillin, streptomycin, sulfamethoxazole and amoxicillin/ clavulanic acid (S1 Table). Only 2.5% (2/79) of *S*. Enteritidis isolates were shown resistance to ampicillin-chloramphenicol-streptomycin-sulfamethoxazole-tetracycline (ACSSuT).

### PFGE genotyping

**Fig 2:**
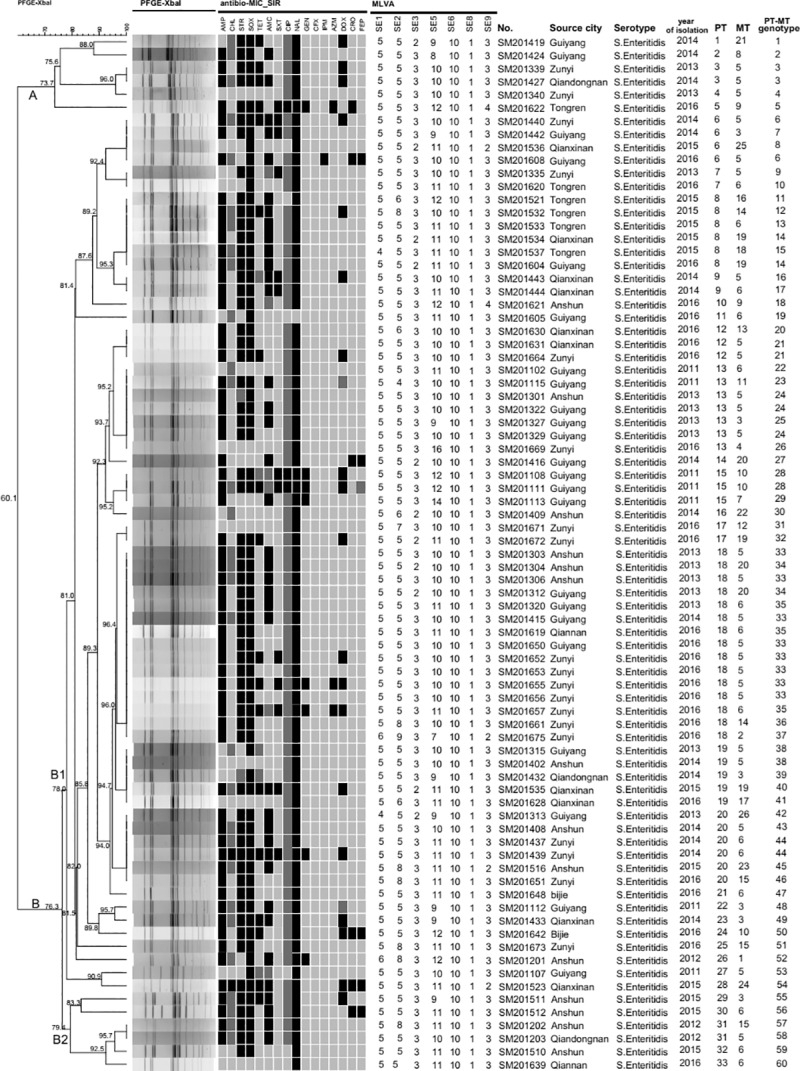
Pulsed-field gel electrophoresis (PFGE) pulsotypes based on *Xba*I enzyme digestion, antimicrobial resistance and multiple-locus variable-number tandem repeat analysis (MLVA) types of 79 *S*. Enteritidis isolates from Guizhou province. A black box represents resistance to an antimicrobial; a dark gray box represents intermediate to an antimicrobial; a light gray box represents susceptibility to an antimicrobial. MLVA types based on seven loci were shown on the right of AMR. The dendrogram was generated by UPGMA. The corresponding background information, pulsotype (PT), MLVA type (MT) and PT-MT genotype were shown on the right of the dendrogram. Genotyping of PFGE combined MLVA (PT-MT genotype) based on PTs and MTs of isolates. If the isolates have the same PTs and the same MTs, isolates are designated genetically indistinguishable.

The 79 *S*. Enteritidis isolates were analyzed by PFGE using enzyme *Xba*I, which generated 33 different *Xba*I pulsotypes (PT1-PT33) with similarity indices ranged from 60.1% to 100% (Fig 2). Among these isolates, 13 pulsotypes were represented by more than one isolate with PT18 (n = 15) containing the most number of isolates, followed by PT13 (n = 7). The dominant PTs were persistent during 2011–2016. All the 79 *S*. Enteritidis isolates were grouped into two clusters (cluster A and cluster B) (Fig 2). The majority of the isolates (n = 73) were grouped in cluster B with 28 pulsotypes (PT6-PT33) and further slip into cluster B1 (n = 67) and cluster B2 (n = 6). The dominant PTs (PT18, PT13, PT8 and PT20) among these isolates grouped in cluster B1. The association analysis among PTs, AMR profiles and geographic location showed that the dominant AMR profiles gathered mainly in cluster B1. However, no correlation was observed between the pulsotypes, AMR profiles and geographic location (Fig 2).

### MLVA genotyping

**Fig 3:**
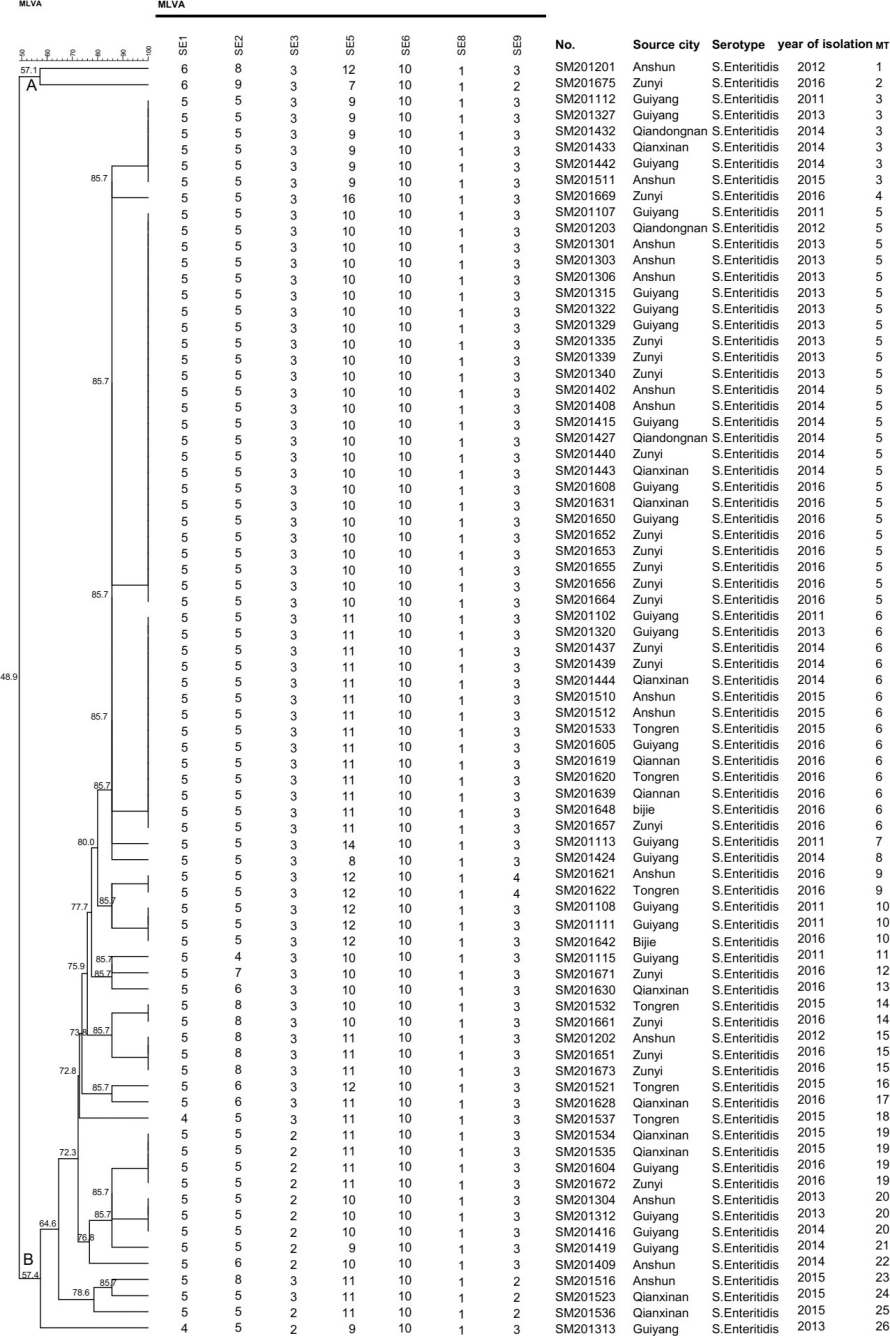
Multiple-locus variable-number tandem repeat analysis (MLVA) types of *S*. Enteritidis isolates from Guizhou province. The dendrogram was generated by UPGMA. The corresponding MLVA type (MT) with copy numbers of the seven loci and background information were shown on the right of the dendrogram.

**Fig 4:**
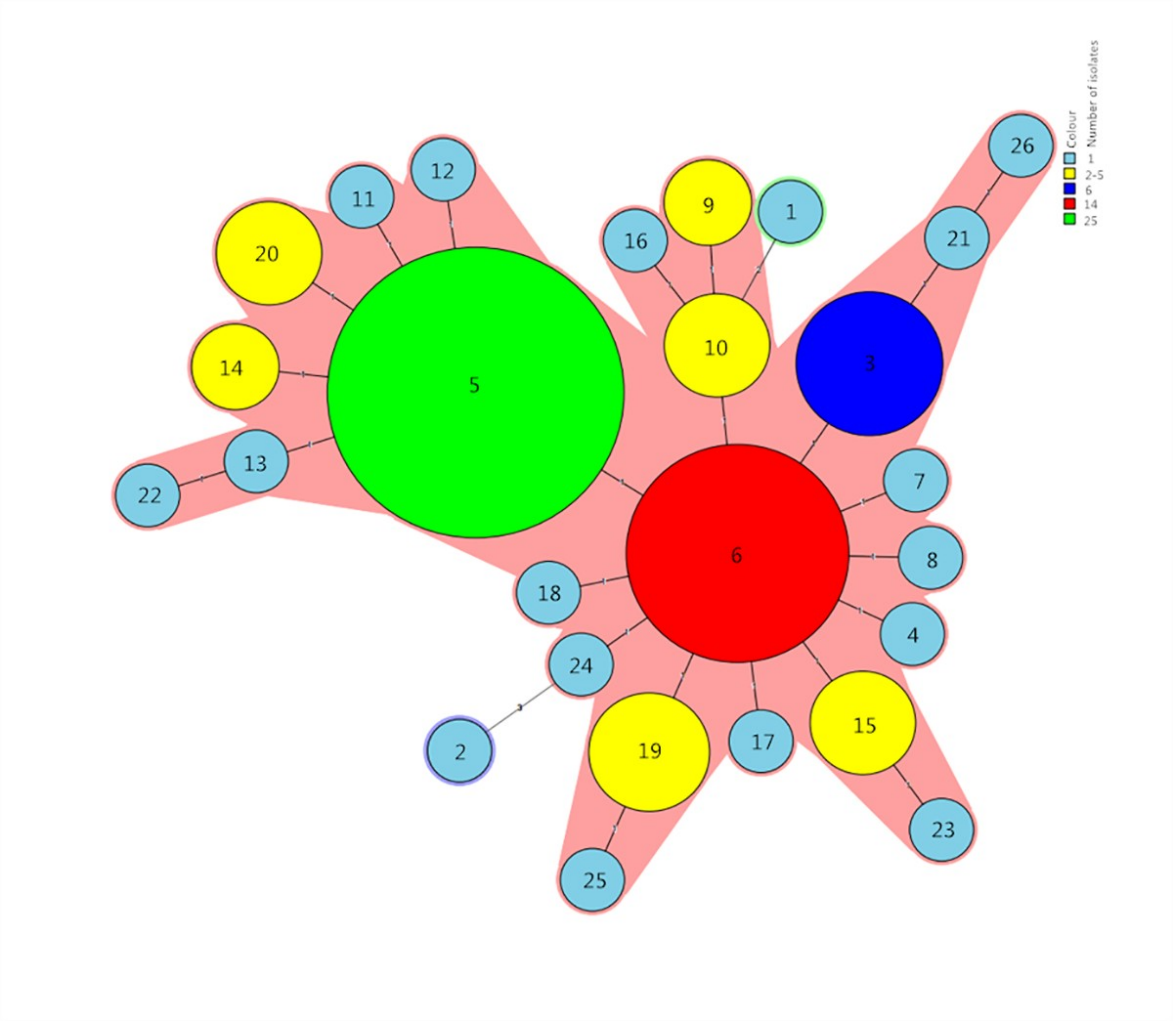
Genetic relationship of 79 *S*. Enteritidis isolates from Guizhou province based on MLVA. The minimum spanning tree (MST) was generated based on 26 MLVA types. Each circle and the number within the circle represent a different MLVA type. The size of the circle is proportional to the amount of the isolates, and the color within the circles represents the different number of isolates. The number outside of the circles indicates how many VNTR loci are different in the MLVA types of connected circles. The shadow zone means these MTs belonging to the same clonal.

MLVA based on seven VNTR loci (SE1-SE2-SE3-SE5-SE6-SE8-SE9) were performed for the characteristics of 79 *S*. Enteritidis isolates further. Among these isolates, 26 different MLVA types (MTs) were discriminated (Fig 3). Nine MTs were represented by more than one isolate with MT5 containing the most number of isolates, followed by MT6 (Figs 3 and 4). MT5 included 25 isolates from 2011 to 2016 except 2015, accounting for 31.6% (25/79) of the total number of isolates. Meanwhile, MT6 contained 14 isolates from 2011–2016 except 2012, accounting for 17.7% (14/79) of the total. Seventeen MTs contained only one isolate. The 79 *S*. Enteritidis isolates were divided into two main clusters, cluster A and B, from the dendrogram generated (Fig 3). A majority (97.4%, 77/79) of isolates were grouped in cluster B with 57.4% similarity. In all isolates, no diversity of the VNTR locus at SE6 and SE8 was observed with tandem repeat numbers of ten and one, respectively. Moreover, the MST of basing on seven VNTR loci of 79 *S*. Enteritidis clinical isolates showed that the MLVA profiles were clustered a single clone with two singletons (Fig 4).

### Genotyping of PFGE combined with MLVA

The genotyping of the Guizhou *S*. Enteritidis isolates were also determined using PFGE combined with MLVA. If the isolates have the same PTs and the same MTs, isolates are designated genetically indistinguishable. The 79 *S*. Enteritidis clinical isolates were divided into 60 unique PT-MT genotypes (Fig 2). Forty-eight PT-MT genotypes contained only one isolate. Twelve PT-MT genotypes were represented by more than one isolate with PT18-MT5 genotype containing the most number of isolates, accounting for 10.1% (8/79) of *S*. Enteritidis isolates. Furthermore, no relationship was observed between the PT-MT genotypes, AMR profiles and geographic location (Fig 2). *S*. Enteritidis isolates from Guizhou showed higher genetic diversity using PFGE combined with MLVA.

## Discussion

*S*. Enteritidis is the most frequent etiological agent of salmonellosis in humans and poultry. The surveillance of *Salmonella* from infectious diarrheal cases indicated *S*. Enteritidis was one of the most important pathogens and the most common serotype in Guizhou province [13, 14]. However, these studies did not provide enough information concerning AMR and molecular epidemiological characteristics of *S*. Enteritidis isolates in Guizhou. Decreased susceptibility to ciprofloxacin and resistance to cephalosporins in *S*. Enteritidis isolates are not reported. To systematically understand the AMR and molecular characteristics, we collected 79 *S*. Enteritidis clinical isolates from Guizhou province in 2011–2016 for analysis in this study.

In the present study, the AMR and MICs of 79 *S*. Enteritidis clinical isolates from Guizhou were detected against 16 antimicrobial agents for the first time here. The results exhibited high-level resistance to antimicrobial agents. All (100%) *S*. Enteritidis isolates were resistant to at least one antimicrobial agent, which was higher than those reported from the Midwestern United States (50%), Spanish (61.7%), Serbia (22%) and Shanghai (77.3%) [23–26]. Furthermore, a high proportion (81.0%) of MDR *S*. Enteritidis isolates in our study was observed (Fig 1), which was significantly higher than the rate of 2.0% (84/4208) from the NARMS report (2006–2015) [27]. The observed MDR prevalence in Guizhou was similar to those reported from eight provinces in China (70.2%) [28]. A similar high prevalence of MDR *S*. Enteritidis was reported in pork (88.46%) and chicken (94.1%) in Guizhou [21, 29]. Meanwhile, a proportion of isolates (44.3%) were observed to be resistant to at least the combination of traditional antibiotics, including ampicillin, streptomycin, sulfamethoxazole and amoxicillin/clavulanic acid in this study (S1 Table). The high-level MDR further limits the option of medicines in the clinical treatment of *S*. Enteritidis infection. Therefore, controlling the application of antibiotics for clinical patients and agriculture was essential, since the antibiotics abuse may further accelerate the accumulation and spread of antimicrobial resistance. More importantly, the routine AMR surveillance of *Salmonella* isolates was crucial to early warn the spread of MDR [30–32].

Ciprofloxacin is recommended to treat salmonellosis as the first-line antibiotic by the World Health Organization [33]. Only 3.8% of isolates were resistant to ciprofloxacin in this study, which was lower than *S*. Enteritidis clinical isolates from Western China (20%), Thailand (51.1%) and Iran (90.9%) [34–36]. However, a great proportion of isolates (92.4%) showed decreased susceptibility to ciprofloxacin (MIC ≥0.12 μg/mL), which was in agreement with other cities in China [37–39]. Moreover, this finding was significantly higher than the rate of 7.2% (303/4208) from the NARM report during 2006–2015 [27]. Due to the decreased susceptibility to ciprofloxacin, the clinical treatment of *S*. Enteritidis infection may lead to treatment failure. Meanwhile, in our study, almost all isolates (except one isolate) were observed resistance to nalidixic acid (Table 1), which was correlated with the decreased susceptibility to ciprofloxacin. Therefore, nalidixic acid susceptibility testing was valuable before using ciprofloxacin for the treatment of *S*. Enteritidis infection in Guizhou.

Moreover, the third-generation cephalosporins were approved to treat salmonellosis [40] and used in livestock and poultry in China [41]. In our study, resistance to the third and fourth-generation cephalosporins had emerged since 2014 in Guizhou (Table 1). Six (7.6%) *S*. Enteritidis isolates exhibited resistance to the third-cephalosporin (ceftriaxone), of which five isolates showed co-resistance to the fourth-generation cephalosporin (cefepime) and decreased susceptibility to ciprofloxacin (S1 Table). If these isolates are globally prevalent, it might become a remarkable public health concern. Therefore, monitoring the dynamic change of resistance to fluoroquinolones and cephalosporins of *S*. Enteritidis isolates was essential. Meanwhile, these data further highlight the necessity to manage the use of clinical antibiotics appropriately.

PFGE genotyping can be used to ascertain the homology of the same serotype isolates [26]. Genetic diversity among *S*. Enteritidis isolates from Guiyang city in Guizhou province had reported. Eleven *S*. Enteritidis clinical isolates were divided into eight PFGE genotypes with *Xba*I [14]. In our study, PFGE analysis revealed 33 different PTs with 60.1% similarity, which showed high genetic diversity and indicated that it was a useful tool in discriminating *S*. Enteritidis isolates. Notably, cluster B was the primary PFGE type of *S*. Enteritidis in Guizhou. There were dominant PTs among the *S*. Enteritidis clinical isolates in Guizhou. However, PFGE pulsotypes were not a correlation with AMR profiles and geographic location. This finding implied that these patients were most likely sporadic. We found a few dominant PFGE pulsotypes during 2011–2016, which implicated these common pulsotypes were persistent (Fig 2).

MLVA had proven to be a reliable method for the molecular epidemiological investigation of *S*. Enteritidis outbreaks and the surveillance of *S*. Enteritidis [42, 43]. In our study, the 79 *S*. Enteritidis isolates were divided into 26 distinct MTs with 48.9% similarity based on seven VNTR loci, which showed a high discriminatory power. A similar study demonstrated that MLVA had a high discriminatory power for *S*. Enteritidis in China [44]. Furthermore, the dominant MLVA types of *S*. Enteritidis clinical isolates were persistent, which was similar to the mid-east of China [45]. The phylogenetic tree of MLVA types showed no correlation was observed between MLVA types and geographic location, which further indicated that these isolates came from sporadic cases. Similar to previous reports [42, 44], all of *S*. Enteritidis isolates had the same number of the tandem repeat at locus SE6 and SE8 in this study (Fig 3). Meanwhile, the VNTR locus SE5 had a high diversity based on seven VNTR loci. This result indicated that the VNTR locus SE6 and SE8 maybe not be detected, as no diversity was found in our study. Further analysis of the MST based on seven VNTR loci showed 79 *S*. Enteritidis clinical isolates belonged to a single MLVA cluster with two singletons, which indicated that these isolates had a closer genetic relationship and came from the same clone (Fig 3).

Analysis of PFGE combined with MLVA showed that 79 *S*. Enteritidis clinical isolates were divided into 60 unique PT-MT genotypes (Fig 2), which revealed higher diversity than single PFGE or MLVA analysis. No epidemiological correlation was observed among these *S*. Enteritidis clinical isolates, which further proved that these isolates came from sporadic cases (Fig 2). The genotyping of *S*. Enteritidis isolates using PFGE combined with MLVA was superior to using a single PFGE or MLVA method, which can provide crucial epidemiological information. Furthermore, it is useful to the investigation of outbreak and epidemiological surveillance of *S*. Enteritidis infection. MLVA can improve public health surveillance of *S*. Enteritidis and has been proposed as a supplement to PFGE for subtyping *S*. Enteritidis [8, 46].

Although the genotyping of PFGE and MLVA can provide important molecular epidemiological information for *S*. Enteritidis isolates from Guizhou province, We do not know if these *S*. Enteritidis isolates belong to the same clone with isolates from other counties or continents. It would be of crucial importance to further learn the whole gene sequence (WGS) to prevent and control *S*. Enteritidis infection in our future studies, which would provide multiple genetic data, including revealing genetic relatedness with isolates from other counties or continents, helping with source trace-back investigations and predicting antimicrobial resistance.

## Conclusion

This study investigated the AMR, PFGE and MLVA molecular genotyping of 79 *S*. Enteritidis clinical isolates from 2011 to 2016 in Guizhou, China. High-level resistance to antimicrobials was observed. All isolates were resistant to at least one antimicrobial. A high burden of MDR was found. Co-resistance to the third and fourth-generation cephalosporins and a high proportion of decreased susceptibility to ciprofloxacin exhibited. It is essential to control the abuse of antimicrobial agents and strengthen the surveillance of AMR. PFGE analysis with *Xba*I enzyme digestion divided the 79 *S*. Enteritidis isolates into 33 different PTs and MLVA analysis of these isolates based on seven VNTR loci obtained 26 distinct MTs.

Analysis of PFGE combined with MLVA for 79 *S*. Enteritidis isolates revealed higher diversity than single PFGE or MLVA analysis. No correlation was observed between these genotypes, AMR profiles and geographic location. The genotyping of *S*. Enteritidis using PFGE combined with MLVA can provide crucial epidemiological information. To our knowledge, this is the first study that we reported the AMR and the molecular genotyping of *S*. Enteritidis clinical isolates from Guizhou province of Southwestern China. These results might be useful to provide a scientific basis for control and prevention of salmonellosis in Guizhou province.

## Supporting information

S1 Table The information of 79 *S*. Enteritidis isolates and the MICs of 16 antimicrobial agents. (XLSX)
